# Combination of Genetic Markers and Age Effectively Facilitates the Identification of People with High Risk of Preeclampsia in the Han Chinese Population

**DOI:** 10.1155/2018/4808046

**Published:** 2018-07-19

**Authors:** Lu Zhou, Xinjie Hui, Huijuan Yuan, Yinglin Liu, Yejun Wang

**Affiliations:** ^1^Shenzhen Maternal and Child Health Care Hospital Affiliated to Southern Medical University, China; ^2^Department of Cell Biology and Genetics, Shenzhen University Health Science Center, China; ^3^Department of Immunology and Internal Rheumatics in Children's Hospital of Anhui Province, China; ^4^Laboratory Department, Shenzhen Maternal and Child Health Hospital Affiliated to Southern Medical University, China

## Abstract

**Objective:**

This study aimed to analyze the possible association between known genetic risks and preeclampsia in a Han Chinese population.

**Methods:**

A total of 156 patients with preeclampsia and 286 healthy Han Chinese women were enrolled and genotyped for 27 genetic alleles associated with preeclampsia in different populations. The association between the genotypes of the individual alleles and preeclampsia and the possible interaction among the alleles were analyzed. Finally logistic models were trained with the genotypes of possible alleles contributing to preeclampsia.

**Results:**

Seven alleles were significantly or marginally significantly associated with preeclampsia, which involved six genes (rs4762 in* AGT*, rs1800896 in* IL-10*, rs1800629 and rs1799724 in* TNFα*, rs2070744 in* NOS3*, rs7412 in* APOE*, and rs2549782 in* ERAP2*). A multilocus interaction analysis further disclosed an interaction among seven alleles. A logistic model showing individual or synergetic contribution to preeclampsia could reach ~0.67 preeclampsia prediction accuracy in the Han Chinese population, while integration of age information could improve the performance to ~0.75 accuracy using a fivefold training-testing evaluation strategy.

**Conclusions:**

The genetic factors were closely associated with preeclampsia in the Han Chinese population despite large ethnicity heterogeneity. The genotypes of different alleles also had synergetic interactions.

## 1. Introduction

Preeclampsia (PE) is a clinical syndrome complicating 2%–8% of pregnancies worldwide. It is a leading cause of maternal and perinatal morbidity and mortality [1, 2]. It is characterized by new-onset hypertension and proteinuria at ≥20 weeks of gestation [3, 4]. PE impairs multiple organs including kidney, liver, and brain, and can cause anasarca, HELLP (hemolysis, elevated liver enzymes, and low platelets) syndrome, cerebral edema, impaired liver or kidney or heart function, abruption of placenta, intrauterine growth restriction, preterm delivery, and even maternal and fetal death [3–5]. Although the underlying mechanisms are generally unknown, PE pathophysiology is closely related to the failure of spiral artery remodeling, impaired extravillous trophoblast invasion, failure of maternal immune tolerance, placental damage by inflammatory stimuli, and dysfunction of maternal vascular endothelium [4–8]. Accumulating evidence has shown the associations between PE and genetic [9–11] and environmental factors [2–4, 12, 13] and their interplays [3, 14].

The early risk prediction could improve the morbidity, mortality, and clinical outcomes of patients with PE [15–17]. The environmental factors, disease history, and concurrent clinical manifestations have been identified as risk factors and used for the clinical guidance of earlier detection of PE [12, 13] despite the critical arguments on the weak specificity and sensitivity [18]. Recently, a couple of genetic factors have been demonstrated to have an association with PE onsets independently or interactively with each other or with environmental factors [9–11, 19, 20]. Inclusion of the high-risk or low-risk genetic factors could improve the prediction sensitivity and specificity of PE, facilitating earlier antenatal screening and intervention of population with higher PE susceptibility [21]. However, studies also disclosed the large variance in genetic components and corresponding PE risk odds among ethnic populations [20, 22, 23]. Therefore, population-specific identification of genetic-risk factors appears necessary and significant for more effective prediction of population with a high or low PE susceptibility.

A previous study explored the genotypes of a Chinese population and showed the different PE risk profile of the putative risk alleles between Chinese and other ethnic groups [23]. Both the sample size and the putative risk allele species were enlarged in this study to generate a more robust and broader PE genetic-risk profile in Chinese women. The confounding factors, such as age, were also investigated. Furthermore, logistic regression models were built to effectively distinguish Chinese people with high or low risks of PE based on the genetic and clinical features.

## 2. Materials and Methods

### 2.1. Participants

A total of 442 unrelated women with first pregnancies (156 with PE and 286 controls) were enrolled at the High-Risk Pregnancy Outpatient Service in Maternal and Children's Hospital of Shenzhen City between June 2014 and May 2015. Diagnosis of PE was defined as new-onset hypertension, combined with proteinuria. Hypertension was defined as systolic blood pressure (BP) ≥140 mm Hg or diastolic BP ≥90 mm Hg. Proteinuria was defined as urinary protein excretion ≥300 mg/24 h or a positive urine dipstick result of at least 1+ without urinary infection [24]. Women in the control group had neither PE in the current pregnancy nor history of previous pregnancies with PE. Participants with multiple gestation, any malformation, any form of hypertension, diabetes mellitus, chronic infectious diseases, autoimmune diseases, thyroid disease, chronic renal disease, rheumatoid arthritis, or systemic lupus erythematosus were excluded from the study. This study was approved by the institutional review board of the Maternal and Children's Hospital of Shenzhen City. Written informed consent was obtained from all participants. All included participants were of Han Chinese origin and lived in the same region at the time of the study.

### 2.2. Single-Nucleotide Polymorphism Selection and Genotyping

In this study, 27 polymorphisms in 19 genes were selected [9, 11], which showed a significant association with PE in populations with different ethnic background and function in coagulation and fibrinolysis, renin-angiotensin system, oxidative stress, inflammation, or lipid metabolism (Table 1).

Genomic DNA was extracted from peripheral blood samples. Except VNTR (Variable Number of Tandem Repeats) in eNos Intron 4 and ACE (angiotensin converting enzyme, ACE) rs4646994 for which genotyping was performed according to the protocols described in previous studies [25, 26] and in Supplementary Materials (available here), the remaining 25 polymorphisms were determined using a multiplex polymerase chain reaction (PCR) reaction and SNaPshot method. Briefly, single-nucleotide polymorphisms (SNPs) were amplified using the KAPA HotStartTaqDNA polymerase (KAPA Biosystems Inc., MA, USA) and further analyzed with single-base extension (SBE) reactions using the SNaPshot Multiplex kit (Applied Biosystems, CA, USA). PCR and SBE primers are listed in Supplementary Materials. The products were purified and analyzed using an ABI3730XL (Applied Biosystems). Twenty individuals were selected randomly for bidirectional sequencing to confirm the accuracy of the genotyping. No genotyping error was observed. The general success rate of genotyping reached 99.7%.

### 2.3. Statistics and Interaction Analysis of Multiple Genetic Features

The chi-square and EBT (exact binomial test) tests were performed in R (https://www.r-project.org/) for rate comparisons as indicated in the context [27]. Multifactor dimensionality reduction (MDR) was used to analyze the interaction among genetic factors [28]. Significance levels were defined as follows:* P *< 0.05, significant; 0.05 ≤* P* < 0.1, marginally significant.

### 2.4. Logistic Regression Modeling of PE Risk with Genetic Markers and Other Features

Genetic markers were reencoded with “1” and “0” for the genotypes with recessive models of major alleles. Each participant in positive (PE) or negative (control) group was represented by a vector of binary digits indicating the genotype composition of the series of SNP markers. The genetic feature matrix of all the participants was imported into R, and logistic regressions were performed with the* glm* function. For the models with both genetic and age features, feature representation for genotype composition was the same as described earlier, while (pregnancy) age was encoded by a 1-bit binary digit: “1” if “>32 years” or “<21 years” and “0” if “≥21 years and ≤32 years.” Each participant in positive (PE) or negative (control) group was represented by a vector of binary digits indicating the genotype composition of both the series of SNP markers and age stratification. When indicated, age could be encoded with the scheme of “1” representing “>40 years” or “<18 years” and “0” representing “≤40 years and ≥18 years.”

Software tools were developed with GO programming language to implement the logistic regression models predicting PE risks (http://www.szu-bioinf.org/PERPer_Go). As described in the document, the genotypes of targeted SNP loci and/or age were used as input, and the output was the probability of PE. Presently, the software is implemented in Linux or Mac operating system.

### 2.5. Performance Assessment of Computational Models on PE Risk Prediction

A fivefold training-testing evaluation strategy was adopted, which was proposed in a previous study to make a fair evaluation on the performance of the computational models with limited samples and genetic data [27]. Briefly, both the PE and control groups were randomly divided into five subgroups. Four of the subgroups were combined for each group and together comprised the training dataset, and the remaining subgroups for patients with PE and controls were combined as the testing dataset. In this way, the original dataset was split into two independent parts, with the training dataset to optimize model parameters and the testing dataset to assess the performance of the model. The splitting, model training, and performance evaluation were repeated five times, and the average performance was calculated over the repeats.

Sensitivity (Sn), specificity (Sp), accuracy (Acc), receiver operating characteristic (ROC) curve, and the area under the ROC curve (AUC) were used to assess the performance of models. In the following formulas, Sn (true positive rate) and Sp (true negative rate), respectively, represented the percentage of positive instances (PE) and the percentage of negative instances (control) correctly predicted. Acc denoted the percentage of both PE and control instances correctly predicted. The ROC curve was a plot of Sn versus (1 − Sp), generated by shifting the decision threshold. The AUC gave the measure of classifier performance. Sn = TP/(TP + FN); Sp = TN/(TN + FP); Acc = (TP + TN)/(TP + FP + TN + FN), where TP, TN, FP, and FN denoted the number of true positives, true negatives, false positives, and false negatives, respectively.

## 3. Results

### 3.1. Different Age Distribution between PE and Control Population

The clinical factors that could possibly contribute to PE risk (e.g., history of PE, smoking, multiple gestation, and concurrent diseases) were controlled strictly during participant recruitment. The only two exceptions included age and BMI. For age, a previous stratification criterion (≤ or >40 years) was adopted, with no difference between groups in the age composition [23, 29]. For all the PE cases recruited in the study, the disease happened not before 34 weeks after pregnancy.

**Figure 1:**
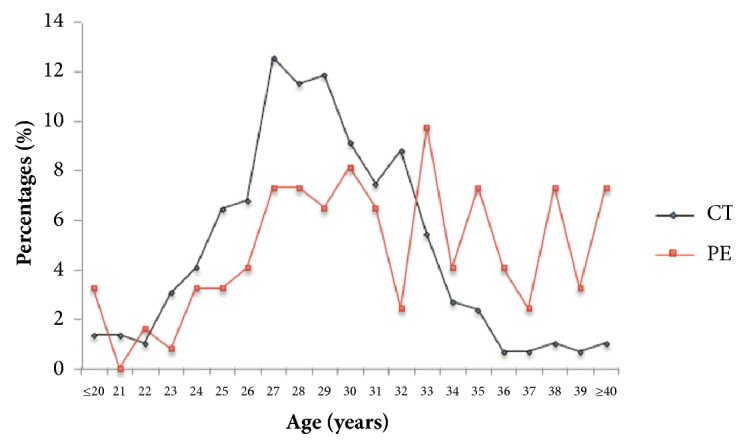
**Difference in age composition between patients with PE and controls.** The percentages of patients with PE (red) and controls (CT, black) at different ages (years) are shown.

The percentages of patients with PE and controls were plotted versus age to further observe the possible relationship between age and PE in Chinese women (Figure 1).

Interestingly, the age of controls showed a clock-shape distribution with a mean of 28–29 years; however, the PE group showed a strikingly different distribution, with an apparent percentage increase on both ends, >32 years and <21 years, and a decrease between 21 and 32 years compared with controls (Figure 1; chi-square test,* P *= 2.34E-12; EBT test,* P* = 4.54E-05). This suggested that age also influenced PE in Chinese population, and the risk stratification levels for age should be adjusted [29].

### 3.2. Different Body Mass Index Distribution between PE and Control Population

**Figure 2:**
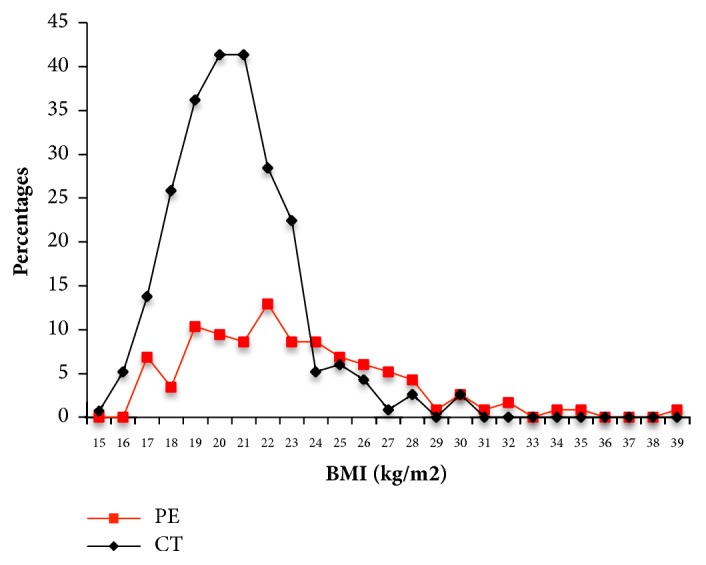
**Difference in BMI distribution between PE patients and controls.** The percentages of patients with PE (red) and controls (CT, black) with different BMIs (kg / m^2^) are shown.

The PE group had a 15-week Body Mass Index (BMI) distribution between 17.22 and 39.74 with a medium of 22.74 kg/m^2^, while the 15-week BMI of control group ranged from 15.62 to 30.86 with a medium of 20.83 kg/m^2^ (Figure 2). The difference between groups was significant, with the higher BMI in PE patients (*P* = 3.56e-9, Mann-Whiney U test).

### 3.3. Ethnicity-Specific PE-Associated Genetic Risks in the Han Chinese Population

The 27 genetic loci with known polymorphisms were genotyped in patients with PE and healthy pregnant women of Han Chinese ethnicity (Table 1). Different from the previous reports on other populations, seven loci showed homozygous allele composition without any polymorphism detected in the Han Chinese patients with PE or controls (rs5742620, rs1799963, rs1799889, rs268, rs4986790, rs4986791, and rs1800590; Table 1). All the remaining 20 loci showed polymorphisms, and the genotype composition followed the Hardy–Weinberg equilibrium (chi-square test,* P *> 0.05). However, 65% (13/20) did not show different allele or genotype composition between PE and control groups (chi-square test,* P *> 0.1, for both allele and genotype comparison; Table 1), further reflecting the ethnic specificity of genetic risks on PE.

Only five loci (rs2070744, rs1800896, rs1800629, rs1799724, and rs4762) showed a significant difference in genotype composition between patients with PE and controls (chi-square test,* P *< 0.05) and additional two (rs2549782 and rs7412) showed marginal significance (*P *< 0.1 and* P *≥ 0.05) (Tables 1 and 2). For these loci, the minor alleles were all consistent with previous reports on different populations (Table 1). The detailed genotype composition and the PE risk odds ratio were calculated for each significant or marginally significant locus based on a recessive model of major alleles (Table 2).

In the Han Chinese population, the genotypes composed of heterozygous or homozygous minor alleles contributed a significantly higher risk to PE in rs2070744 (TC/CC), rs1800896 (AG/GG), and rs1799724 (CT/TT); a marginally higher risk to PE in rs2549782 (TG/GG) and rs7412 (CT/TT); and a protective effect to rs1800629 (GA/AA) and rs4762 (CT/TT) (Table 2).

The genetic polymorphisms appeared to contribute to PE independent of the age factor because the association analysis after the stratification of PE and control groups according to the age distribution shown in Figure 1 yielded similar results (data not shown).

### 3.4. Complex Interactions among Genetic Polymorphisms Associated with PE

The MDR interaction analysis was performed to observe possible interactions among the 20 polymorphic genotypes showing the contribution to PE. Among different combinations, the best model showed a significant and stable interaction among seven polymorphic loci from six genes (Table 3).

**Figure 3:**
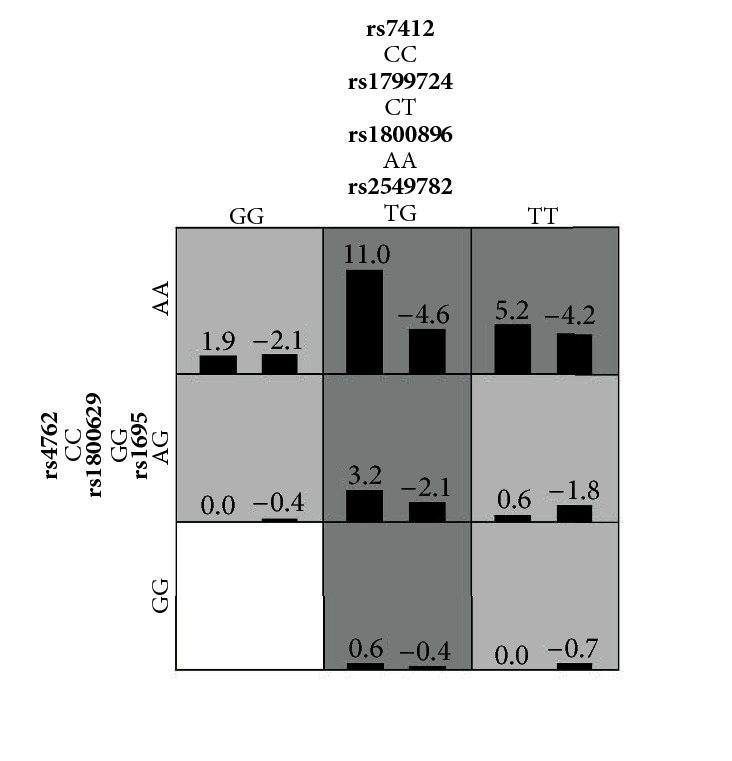
**An example of the contribution of interaction among seven alleles to PE risk.** The positive and negative contributions are shown in dark and light gray, respectively.

The loci included rs2549782 (*ERAP2*), rs1695 (*GSTP1*), rs1800896 (*IL-10*), rs1800629 and rs1799724 (*TNF-alpha*), rs4762 (*AGT*), and rs7412 (*APOE*). The interaction was complex due to the involvement of many loci. However, among the interactions, some synergetic effect could be clearly identified on PE, for example, among the genotype of TG (rs2549782), AA or AG (rs1695), AA (rs1800896), GG (rs1800629), CT (rs1799724), CC (rs4762), and CC (rs7412) (Figure 3).

### 3.5. Genetic Polymorphic Features Could Identify High-PE-Risk Han Chinese Population with Limited Power

A logistic model was trained with the genotypes of eight polymorphic loci (rs2549782, rs1799724, rs1695, rs1800896, rs1800629, rs2070744, rs4762, and rs7412) (Table 4).

Among these loci, rs1800896, rs1799724, rs2070744, rs4762, and rs7412 showed significant contributions in the model to PE risks (Table 4). Consequently, another logistic model was also trained only with the genotype features of the five significant contributing SNPs (Supplementary Table S1).

**Figure 4:**
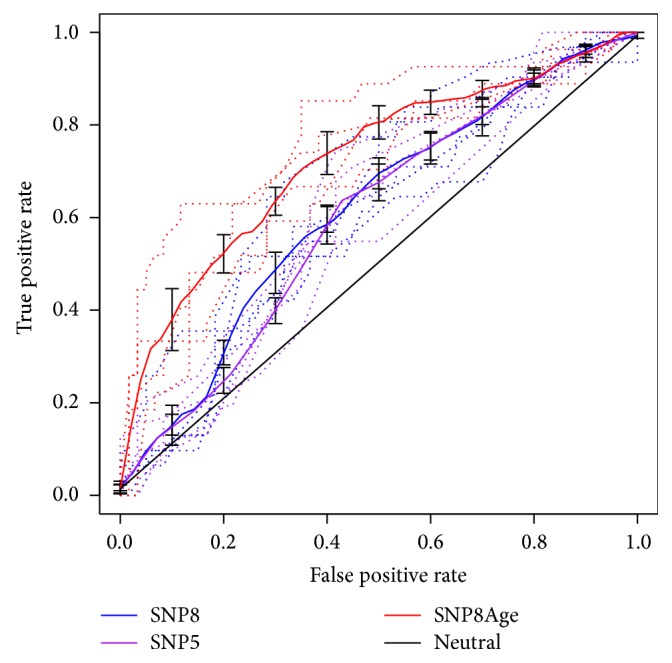
**ROC curves of PE risk prediction models.** For each type of model, the full curve represents the average performance of fivefold training-testing results, while the dashed curves represent individual performance. “Neutral” means the random situation with 50% AUC of ROC curve.

A training-testing strategy was adopted to make a fair assessment on the predictive performance of the logistic models based on genetic features in the Han Chinese population (refer to Materials and Methods). On the basis of the aforementioned eight SNPs, the models could reach an average AUC of 0.618, with optimized accuracy, specificity, and sensitivity of 0.674, 0.789, and 0.465, respectively (Table 5; Figure 4, SNP8).

The performance was apparently better than that of the neutral random model, which showed an AUC of 0.500 (Figure 4, neutral). The models based on five significant contributing SNPs showed slightly lower performance, with average AUC, accuracy, specificity, and sensitivity of 0.603, 0.658, 0.852, and 0.303, respectively (Table 5; Figure 4, SNP5).

### 3.6. Combination of Genotype and Age Information Could Improve the Prediction Power of PE Risks in the Han Chinese Population

At present, age is one of the most important factors for a clinical doctor to evaluate the general risk of PE. Therefore, the genetics-based models were further compared with the simplest age model for predictive power in identifying PEs.

The levels of risk stratification by age are different among different countries or areas, for example, “>32 years” being considered as a high-risk factor in some countries but “>40 years” in others such as China [12, 29]. This study showed the high PE risks of “>32 years” and “<21 years” in the Han Chinese population (Figure 1). Therefore, the age model followed the new age stratification schemes (refer to Materials and Methods). As a result, the pure age model could reach an AUC, accuracy, specificity, and sensitivity of 0.598, 0.633, 0.677, and 0.591, respectively, which were quite close to or slightly worse than the performance of the models based on pure genetic features (SNP5 or SNP8) (Table 5). The age model was also evaluated based on previous stratification levels, that is, “<18 years,” “18–40 years,” and “>40 years”; however, it worked far worse.

The genetic features were further combined with age stratification, and a new logistic model was trained. In the new model, the “age” contributed most significantly while the contribution of different genetic markers varied largely from pure genetic models, indicating the significance of age and the complex interactions between age and genetics in PE (Supplementary Table S2). The training-testing evaluation further demonstrated the strikingly improved prediction power of the model based on both genetic markers and age information (Table 5; Figure 4, SNP age). The average AUC, optimized accuracy, specificity, and sensitivity reached 0.687, 0.749, 0.856, and 0.504, respectively (Table 5).

Other machine learning techniques were also adopted to build prediction models based on the eight or five SNPs, for example, support vector machine with different kernel types. However, based on the training-testing evaluation results, none of them outperformed the logistic models (data not shown).

Besides the age and genetic features, the other clinical feature, BMI, was also evaluated for the performance as a PE predictor. It could predict PE with an average accuracy of 0.69, not as good as the SNP8Age model. Due to the missing of height information for a substantial portion of the PE cases (~15%), BMI was not integrated into the combined model as an individual feature in this research.

## 4. Discussion

Accumulating evidence supported the association between the occurrence and progression of PE and genetics [9, 11]. On the contrary, the ethnicity heterogeneity for the association was observed repeatedly [20, 22, 23]. This study further observed the ethnicity heterogeneity in the Han Chinese population for the genetic risks of PE. All the investigated 27 alleles were reported with polymorphisms previously, which were associated with PE in different populations. However, seven of them showed homogeneity without polymorphism among all the 442 Han Chinese participants (Table 1). For the other 20 alleles, 13 did not show any association between PE and allele composition or genotype (Table 1). Even for the remaining alleles with significant or marginally significant risk for PE in the Han Chinese population, the ethnicity difference was still observed. For example, rs4762 was previously shown with a polymorphism in a Korean cohort, but different genotypes did not show a significant bias between patients with PE and controls [30]. For rs1800896, an active debate continues on the association and the risk PE allele composition (A or G) in different populations [31–35]. No association was frequently observed in multiple populations for rs1800629 [36, 37], while the association and risk PE genotype (AA or TT) remain contradictory for rs1799724 [38, 39]. Despite the ethnicity difference disclosed in this study, the project is still ongoing with an enlarged sample size. Moreover, the possibility could not be excluded that some of the alleles shown without polymorphism or no association with PE could show association with PE with the increase in size.

Seven alleles were found in the Han Chinese population significantly or marginally significantly associated with PE (Tables 1 and 2). The genes involved included* AGT*,* IL-10*,* TNF-alpha*,* NOS3*,* APOE* (marginally), and* ERAP2* (marginally) (Table 2). The* AGT* gene product is an important component of the rein-angiotensin system (RAS), a key regulatory system of blood pressure, which could be closely related to PE occurrence [40]. The PE risk allele in rs4762 of* AGT* gene in the Han Chinese population could cause amino acid change (T174 M).* IL-10* and* TNF-alpha* are inflammation-related genes that participate in anti-inflammatory responses observed in PE [41]. In this research, one allele in* IL-10* promoter (rs1800896) and two alleles in* TNF-alpha* promoter (rs1800629 and rs1799724) were found to be associated with PE. The allele “G” and genotypes “GG”/”AG” were associated with a high risk for PE in rs1800896. The composition of allele “A” and genotypes “AA”/”AG” significantly decreased while “T” and genotypes “TT”/”CT” significantly increased in PE for rs1800629. The other genes were also reported to be associated with the occurrence or progression of PE. However, the underlying molecular mechanisms remain unclear and require further investigation. Besides the individual contribution to PE, the alleles (or genes) also showed a complex interaction (Figure 2). More samples are needed to definitively confirm the synergetic action among the alleles before the experimental exploration of the molecular mechanism. It should also be noted that all the PEs in the study occurred in the later stage of pregnancy (>34 weeks), and therefore the maternal factors likely had more significant roles. However, it would still be interesting to further examine the genotypes of the paternal side and their possible contribution.

This study developed regression models based on the association between genotype features and PE in the Chinese population, which could predict the risk of PE. Notably, the models simply based on genetic features identified in the present study only showed limited prediction power. However, the performance was still close to or slightly better than the age-based prediction (Table 5). Therefore, genetic features could be considered as important factors as age or other clinical factors when women were screened for PE risk because strategies were urgently desired but still lacked PE screening at an earlier time before or during pregnancy [12, 29]. The genetic features were also combined with age in a new model, which exhibited much better PE prediction power. Therefore, both the genetic features and age should contribute to PE in different ways. The contributions of individual genetic loci in the combined model appeared different from those in the sole genetic models, also suggesting complicated interactions between the genetic features and age in PE (Table 4; Table S2). Also, a software tool, PERPer_Go, was developed, which provided an easy way to implement the genetic (SNP8 or SNP5) and combined (SNP8age) models and predict the PE risk of participants (http://www.szu-bioinf.org/PERPer_Go). This probably is the first application of PE prediction with combined genetic and age features in specific populations. In practice, a high specificity (e.g., 95%) should be controlled, leading to a relatively low PE recalling rate (38.2% for SNP8 age at 95% specificity). Efforts continue to improve the prediction performance of the models in different ways. An enlarged cohort of patients with PE and controls has been recruited and sampled for* de novo* risk genotype detection with whole-genome SNP arrays. Factors other than genetics or age have also been combined for consideration. For example, the association of multiple fetuses and fetus number with PE was observed, revealing that the model with integrated features of genetics, age, and fetus could reach ~44% sensitivity when the specificity was controlled at 95%. Clinical factors, such as PE history and concurrent disorders during pregnancy, are also important and can improve the PE prediction power strikingly. In the current study, we also noticed that PE patients showed significant higher BMI than control. Maternal overweight and obesity have been considered as risk factors for preeclampsia. Despite the single BMI predictor could only reached a ~ 60% accuracy in our dataset, it would still be interesting to integrate the feature in new prediction models in the future.

## 5. Conclusion

PE has a strong genetic factor in its causes and shows a complex process of the interactions of various factors. The MDR model may be an effective method for estimating risks of PE.

## Figures and Tables

**Table 1:** SNPs included in the study and their allele contribution to PE.

**Gene**	**SNP**	**Reported PE minor allele**	**Minor PE allele in the study** ^**1**^
*ACE*	rs4646994	Del	Not sig.
*AGT*	rs699	C	Not sig.
	rs4762	T	T
*APOE*	rs429358	C	Not sig.
	rs7412	T	T
*ATIR*	rs5186	C	Not sig.
*CTLA4*	rs231775	G	Not sig.
*EPHX1*	rs1051740	C	Not sig.
*ERAP2*	rs2549782	G	G
*F2*	rs1799963	A	—
*FV*	rs602	A	Not sig.
	rs6025	A	Not sig.
*GSTP1*	rs1695	G	Not sig.
*IGF1*	rs5742620	A	—
*IL-10*	rs1800896	G	G
*LPL*	rs1800590	G	—
	rs268	G	—
*MTHFR*	rs1801133	T	Not sig.
*NOS3*	27bp-VNTR in intron 4	4a	Not sig.
	rs2070744	C	C
	rs1799983	T	Not sig.
*SERPINE1*	rs1799889	G	—
*TLR4*	rs4986790	G	—
	rs4986791	T	—
*TNF-alpha*	rs1800629	A	A
	rs1799724	T	T
*VEGF*	rs3025039	T	Not sig.

^1^The minor allele at the locus without polymorphism is represented with ‘—'; not significant allele or genotype composition difference between patients with PE and controls is indicated with “not sig.” (chi-square test, *P* ≥ 0.1). For significant (*P* < 0.05) or marginally significant (*P* ≥ 0.05 and < 0.1) ones, the PE-contributing minor alleles are shown.

**Table 2:** Genotype contribution to PE in the Han Chinese population.

**SNP** ^**1**^	**Genotype**	**PE#**	**CT#**	**PE_Major**%	**CT_Major**%	**Chi-square**	***P* value**	**OR**	**95**%**_Upper**	**95**%**_Lower**
rs2549782^2^	TT	42	99	0.27	0.35	2.83	0.09	0.69	1.06	0.45
	TG	88	134							
	GG	26	52							
rs1695^2^	AA	124	211	0.79	0.74	1.79	0.18	1.38	2.20	0.86
	AG	26	67							
	GG	6	8							
*rs2070744*	TT	149	284	0.96	0.99	7.26	0.01	0.15	0.73	0.03
	TC	6	2							
	CC	1	0							
*rs1800896*	AA	120	248	0.77	0.87	6.29	0.01	0.53	0.87	0.32
	AG	32	32							
	GG	3	6							
*rs1800629* ^2^	GG	139	235	0.89	0.82	3.73	0.05	1.77	3.19	0.99
	GA	16	47							
	AA	1	4							
*rs1799724*	CC	92	205	0.59	0.72	7.39	0.01	0.57	0.86	0.38
	CT	61	78							
	TT	3	3							
*rs4762*	CC	133	219	0.85	0.77	4.69	0.03	1.77	2.98	1.05
	CT	23	62							
	TT	0	5							
rs7412^2^	CC	129	252	0.83	0.88	2.81	0.09	0.63	1.09	0.36
	CT	27	32							
	TT	0	1							

^1^The SNP loci showed significant (chi-square test, *P* < 0.05; indicated in italic) or marginally significant (chi-square test, *P* ≥ 0.05 and < 0.1) difference in genotype composition between PE and control groups, except rs1695, for which the difference was not (marginally) significant but showed contribution to PE in multilocus interaction analysis and prediction models. ^2^The 90% confidence interval (upper and lower) for the OR of these SNPs was also calculated: 0.88 and 0.57 for rs2549782, 1.97 and 1.01 for rs1695, 3.01 and 1.22 for rs1800629, and 0.90 and 0.49 for rs7412, respectively. PE# and CT#, the number of patients with PE and controls, respectively; PE_ and CT_major#, the number of patients with PE and controls with the major genotype, respectively; OR, odd ratio; 95%_upper and lower, the 95% upper and lower confidence limits, respectively.

**Table 3:** Interaction among multiple polymorphic loci.

**Model** ^**1**^	**Training Bal. Acc**	**Testing Bal. Acc.**	**CV consistency**	**Sign test (*P*)**
rs1799724	0.5639	0.5645	10/10	7 (0.1719)
rs1800896 and rs1799724	0.5978	0.5592	9/10	8 (0.0547)
rs1800896, rs1800629, and rs1799724	0.6163	0.5196	4/10	6 (0.3770)
rs2549782, rs1695, rs1800896, and rs1799724	0.6391	0.5052	3/10	6 (0.3770)
rs2549782, rs1695, rs1800896, rs1799724, and rs4762	0.6714	0.5362	8/10	7 (0.1719)
rs2549782, rs1695, rs1800896, rs1800629, rs1799724, and rs4762	0.7030	0.5691	8/10	8 (0.0547)
**rs2549782, rs1695, rs1800896, rs1800629, rs1799724, rs4762, and rs7412**	**0.7294**	**0.5853**	**10/10**	**10 (0.0010)**
rs2549782, rs1695, rs2070744, rs1800896, rs1800629, rs1799724, rs4762, and rs7412	0.7378	0.5633	10/10	8 (0.0547)

^1^Only the best interaction models with no larger than eight features are shown. The significant and best model is shown in bold.

“Training Bal. Acc,” training balanced accuracy; “Testing Bal. Acc,” testing balanced accuracy; “CV consistency,” 10-fold cross-validation consistency.

**Table 4:** Contribution to PE risks in the Han Chinese population based on the logistic model of eight SNPs.

Variable	Coefficient	Std. error	z value	Pr(>|z|)	Sign.
Intercept	1.9932	1.0322	1.931	0.05348	.
rs2549782	–0.3618	0.2291	–1.579	0.11435	
rs1695	0.2951	0.2496	1.182	0.23718	
rs2070744	–-2.03	0.8283	–2.451	0.01425	*∗*
rs1800896	–0.7913	0.2715	–2.914	0.00357	*∗∗*
rs1800629	0.4985	0.3116	1.6	0.10963	
rs1799724	–0.6314	0.2193	–2.879	0.00399	*∗∗*
rs4762	0.5468	0.2761	1.98	0.04765	*∗*
rs7412	–0.6265	0.2937	–2.133	0.03291	*∗*

*∗P* < 0.05;* P *< 0.1.

*∗∗P* < 0.01.

*∗∗∗P* < 0.001.

**Table 5:** Average performance of different models based on training-testing evaluations.

**Model**	**Features**	***Sn***	***Sp***	***Acc***	**AUC**
SNP8	Eight SNPs	0.465	0.789	0.674	0.618
SNP5	Five SNPs	0.303	0.852	0.658	0.603
Age	Age	0.591	0.677	0.633	0.598
SNP8Age	Eight SNPs, Age	0.504	0.856	0.749	0.687

## Data Availability

The data used to support the findings of this study are available from the corresponding author upon request.

## Supplementary Materials

Supplementary Materials Supplementary Table S1: contribution to PE risks in the Han Chinese population based on the logistic model of five SNPs. Supplementary Table S2: contribution to PE risks in the Han Chinese population based on the logistic model of eight SNPs and age information. Supplementary Table S3: PCR primer sequences for three multiplex reactions. Supplementary Table S4: extension primer sequences for three multiplex reactions. Supplementary Figure S1: the full interaction among seven alleles to PE risk. The positive and negative contributions are shown in dark and light gray, respectively.
