# Supplementary material for: Combination of Genetic Markers and Age Effectively Facilitates the Identification of People with High Risk of Preeclampsia in the Han Chinese Population
Source: Biomed Res Int. 2018 Jul 19;2018:4808046. doi: 10.1155/2018/4808046 (PMC6077688; doi:10.1155/2018/4808046)
Supplement: Supplementary Materials — Supplementary Table S1: contribution to PE risks in the Han Chinese population based on the logistic model of five SNPs. Supplementary Table S2: contribution to PE risks in the Han Chinese population based on the logistic model of eight SNPs and age information. Supplementary Table S3: PCR primer sequences for three multiplex reactions. Supplementary Table S4: extension primer sequences for three multiplex reactions. Supplementary Figure S1: the full interaction among seven alleles to PE risk. The positive and negative contributions are shown in dark and light gray, respectively. [file 4808046.f1.doc]

**Supplementary Methods**

**1. DNA Genotyping of eNOS Intron 4**

Screening for the VNTR in intron 4 of the eNOS（ endothelial nitric oxide synthase） gene was determined by PCR using the following oligonucleotide primers: forward, 5′-AGGCCCTATGGTAGTGCCTT-3′, and reverse, 5′-TCTCTTAGTGCTGTGGTCAC-3′, which flank the region of the 27-bp repeats in intron 4, as described previously^5^. Reactions were performed in a total volume of 50 μL containing 1 μg genomic DNA, 40 pmol each primer, and 25 μL of PCR Master Mix (2×) (Fermentas, Life Science, Waltham Massachusetts, USA ). The thermocycling proced ure was performed using a PTC-100 (MJ Research, Waltham, Massachusetts, USA) and consisted of initial denaturation at 94°C for 4 min, 35 cycles of denaturation at 94°C for 1 min, annealing at 56°C for 1 min, extension at 72°C for 2 min, and a final extension at 74°C for 7 min. The PCR products were analyzed using 2% agarose gel electrophoresis and visualized using Goodview Nucleic Acid stain (cat: HGV-II).

**2. DNA Genotyping of ACE rs4646994**

ACE rs4646994 polymorphism was genotyped by two PCRs using three primers.^7^ The primers of first PCR were 5’-CTGGAGACCACTCCCATCCTTTCT-3’ and 5’-GATGTGGCCATCACATTCGTCAGAT-3’. The 190-bp and 490-bp products of this PCR were from D and I alleles, respectively. The first amplification reaction was carried out in a total volume of 50 μL, using 2 μg genomic DNA, 40 pmol each primer, and 25 μL of PCR Master Mix (2×) (Fermentas, Life Science). The PCR conditions were 94°C for 5 min, followed by 30 cycles at 94°C for 1 min, 58°C for 1 min, 72°C for 2 min, and a final step at 72°C for 4 min. A second PCR was performed with the same antisense primer as in the first and different sense primer as 5-TTT GAG ACG GAG TCT CGC TC-3, which generated a 408-bp fragment only in the presence of the I allele, to avoid the misidentification of DI genotypes as DD. The second PCR was carried out in a total volume of 50 μL with the same reagents of the first amplification reaction. PCR conditions were 94°C for 5 min, followed by 40 cycles at 94°C for 1 min, 60°C for 75 s, 72°C for 1 min, and a final step at 72°C for 10 min. The PCR products were analyzed using 2% agarose gel electrophoresis and visualized using Goodview Nucleic Acid stain (cat: HGV-II).

Supplementary Table S1. Contribution to PE risks in the Han Chinese population based on the logistic model of five SNPs

| **Variable** | **Coefficient** | **Std. error** | **z value** | **Pr(>|z|)** | **Sign.** |
| --- | --- | --- | --- | --- | --- |
| Intercept | 2.6667 | 0.9647 | 2.764 | 0.00571 | ** |
| rs2070744 | –2.0713 | 0.8198 | –2.527 | 0.01152 | * |
| rs1800896 | –0.7849 | 0.2689 | –2.919 | 0.00351 | ** |
| rs1799724 | –0.6597 | 0.2167 | –3.045 | 0.00233 | ** |
| rs4762 | 0.5193 | 0.2743 | 1.893 | 0.05836 | . |
| rs7412 | –0.6917 | 0.2915 | –2.373 | 0.01765 | * |

**P* < 0.05, *P* < 0.1.

***P* < 0.01.

****P* < 0.001.

Supplementary Table S2. Contribution to PE risks in the Han Chinese population based on the logistic model of eight SNPs and age information

| **Variable** | **Coefficient** | **Std. error** | **z value** | **Pr(>|z|)** | **Sign.** |
| --- | --- | --- | --- | --- | --- |
| Intercept | –1.24966 | 1.49974 | –0.833 | 0.4047 |  |
| rs2549782 | –0.58833 | 0.26958 | –2.182 | 0.02908 | * |
| rs1695 | 0.31971 | 0.28271 | 1.131 | 0.25811 |  |
| rs2070744 | 0.08276 | 1.31952 | 0.063 | 0.94999 |  |
| rs1800896 | –0.71685 | 0.30459 | –2.353 | 0.0186 | * |
| rs1800629 | 0.9317 | 0.40431 | 2.304 | 0.0212 | * |
| rs1799724 | –0.77403 | 0.24881 | –3.111 | 0.00186 | ** |
| rs4762 | 0.45439 | 0.31052 | 1.463 | 0.14338 |  |
| rs7412 | –0.40907 | 0.34877 | –1.173 | 0.24083 |  |
| Age | 1.64441 | 0.25767 | 6.382 | 1.75E-10 | *** |

* *P* < 0.05, *P* < 0.1*.*

** *P* < 0.01.

****P* < 0.001.

**Supplementary Table S3. PCR primer sequences for three multiplex reactions**

| SNP-ID | Gene | Primer |
| --- | --- | --- |
| **Multiplex 1** | | |
| rs1799983 | NOS3 | GGAGATGAAGGCAGG(A/G)GACA |
|  |  | CAGTCAATCCCTTTGGTGCT |
| rs5742620 | IGF1 | GGAATCGTGGGAGTCAATGC |
|  |  | CGAACTGAAGAGCATCCACCA |
| rs2549782 | ERAP2 | ACCTGGTCACAATGGAATGG |
|  |  | ACCTTCAATGTTTCATCCAGTTC |
| rs1695 | GSTP1 | CTGTGTGGCAGTCTCTCATCC |
|  |  | GTTGATGTCCCAGGCAATAAG |
| rs1051740 | EPHX1 | GCTTCCA(C/G)TATGGCTTCAACTC |
|  |  | CTATAATTGGGTTCTGAATCTCTCC |
| rs699 | AGT | GAAGATTGACAGGTTCATGCAG |
|  |  | ACTAAGTCCTAGGGCCAGAGC |
| rs6020 | F5 | TGAACCCACAGAAAATGATGC |
|  |  | CCCCATTATTTAGCCAGGAGAC |
| rs6025 |  | TGAACCCACAGAAAATGATGC |
|  |  | CCCCATTATTTAGCCAGGAGAC |
| **Multiplex 2** | | |
| rs2070744 | NOS3 | ACCAGGGCATCAAGCTCTTC |
|  |  | CCTTGAGTCTGACATTAGGGTATC |
| rs1799963 | F2 | GTATCAAA(T/C)GGGCAT(C/T)GTCTC |
|  |  | CAGAGAGCTGCCCATGAATAG |
| rs1799889 | SERPINE1 | TCCAACCTCAGCCAGACAAG |
|  |  | TCCGATGATACACGGCTGAC |
| rs1801133 | MTHFR | GTCATCCCTATTGGCAGG(T/G)TAC |
|  |  | AGGAAGAACTCAGCGAACTCAG |
| rs4986791 | TLR4 | AAGGCTTACTTTCACTTCC(A/G)ACA |
|  |  | CAAG(G/T)TAAATGAGGTTTCTGAGTG |
| rs3025039 | VEGFA | CCTCAGATGTGACA(A/G)GCCGA |
|  |  | GGTGGGTGTGTCTACA(G/C)GAATC |
| rs231775 | CTLA4 | TGAAGACCTGAAC(A/G)CCGCTC |
|  |  | GAATACAGAGCCAGCCAAGC |
| rs1800896 | IL10 | ACACTACTAAGGCTTCTTTGGGA |
|  |  | TACAAGGGTACACCAGTGC(C/T)A |
| rs268 | LPL | TGAGTTGTAGAAAGAACCGCTG |
|  |  | TACCTTTGTAGGGCATCTGAGA |
| rs4986790 | TLR4 | GGTCTGGCTGGTTTAGAAGTCC |
|  |  | CCCTTTCAATAGTCACAC(T/C)CACC |
| rs1800629 | TNF-alpha | CCCCTCCCAGTTCTAGTTCTATC |
|  |  | TAGGACCCTGGAGGCTGAAC |
| rs1799724 |  | ACCACAGCAATGGGTAGGAG |
|  |  | GGTCCTGGAGGCTCTTTCAC |
| **Multiplex 3** | | |
| rs1800590 | LPL | GGCAGGGTTGATCCTCATTAC |
|  |  | CCAAGGCTGCTTATG(T/A)GACTG |
| rs4762 | AGT | GCTGACAGGCTACAGGCAATC |
|  |  | CATCCAG(T/C)TCT(G/T)TGAAGTCCAGAG |
| rs7412 | APOE | CGGAACTGGAGGAACAACTGAC |
|  |  | TGCTCCTTCACCTCGTCCAG |
| rs429358 |  | CGGAACTGGAGGAACAACTGAC |
|  |  | TGCTCCTTCACCTCGTCCAG |
| rs5186 | AGTR1 | GAGAA(C/T)ATTCCTCTGCAGCACT |
|  |  | AGGGAGATTGCATTTCTGTCAGT |

**Supplementary Table S4.** Extension primer sequences for three multiplex reactions

| **SNP-ID** | **Sequence** | | | | |  |
| --- | --- | --- | --- | --- | --- | --- |
|  |  | **Multiplex 1** | |  |  | |
| rs1799983 | CTGCTGCAGGCCCCAGATGA | | | | |  |
| rs5742620 | AGGATCAAGGAGGAAACCTGA | | | | |  |
| rs2549782 | GGTGGAATGATATTTGGCTTAA | | | | |  |
| rs1695 | AGGACCTCCGCTGCAAATAC | | | | |  |
| rs1051740 | CAAT(C/T)TTAGTC(T/C)TGAAGTGAGGGT | | | | |  |
| rs699 | GAAGACTGGCTGCTCCCTGA | | | | |  |
| rs6020 | TACAGTGACGTGGACATCATGA | | | | |  |
| rs6025 | AGCAGATCCCTGGACAGGC | | | | |  |
|  |  | **Multiplex 2** | |  |  | |
| rs2070744 | GGCTGAGGCAGGG(T/C)CAGCC | | | | |  |
| rs1799963 | TTCCCAATAAAAGTGACTCTCAG(C/T) | | | | |  |
| rs1799889 | TCCGATGATACACGGCTGAC | | | | |  |
| rs1801133 | GCTGCGTGATGA(T/C)GAAAT(C/T)G | | | | |  |
| rs4986791 | TTCTCAAAGTG(A/T)TTTTGGG(A/G)CAA | | | | |  |
| rs3025039 | ATCCAATTCCAAGAGGGAC(C/T) | | | | |  |
| rs231775 | AGTGCAGGGCCAGGTCCTGG | | | | |  |
| rs1800896 | CACTACTAAGGCTTCTTTGGGA | | | | |  |
| rs268 | TGCTTCTTTTGGCTCTGACTTTA | | | | |  |
| rs4986790 | ATACTTA(G/A)ACTACTACCTC(G/A)ATG | | | | |  |
| rs1800629 | CAATAGGTTTTGAGGGGCATG | | | | |  |
| rs1799724 | CCTCTACATGGCCCTGTCTT(C/T) | | | | |  |
|  | **Multiplex 3** | |  | | | |
| rs1800590 | CAAACGTTTAGAAGT(G/C)AATTTA(G/T)G | | | | |  |
| rs4762 | ATCATAAGTTGGGGGGAGGG | | | | |  |
| rs7412 | GCCTGGTACACTGCCAGGC | | | | |  |
| rs429358 | CGCGGACATGGAGGACGTG | | | | |  |
| rs5186 | AGCACT(T/G)CACTACCAAATGAGC | | | | |  |
